# Older adults’ perceptions of wearable technology hip protectors: implications for further research and development strategies

**DOI:** 10.1080/17483107.2018.1491647

**Published:** 2018-10-29

**Authors:** Alex Hall, Elisabeth Boulton, Emma Stanmore

**Affiliations:** Division of Nursing Midwifery & Social Work School of Health Sciences, University of Manchester, Manchester, UK

**Keywords:** Hip protectors, falls, wearable technology, sensor technology, patient and public involvement

## Abstract

**Purpose:** Hip fractures are an important public health issue. Ninety-five percent of hip fractures are caused by falls, with those at greatest risk including residents of long-term care facilities. Hip protectors can be effective in preventing hip fractures, but levels of acceptance and adherence may be low. We report on work to develop research into a new hip protector that aims to overcome some of the acceptance and adherence challenges.

**Methods:** We held five patient and public consultation events involving 147 older adults and 10 long-term care sector staff in the Midlands and North West of England. At each event, participants were shown the Fall-Safe Assist hip protector, which includes built-in mobile technology to record information about falls and summon help from caregivers.

**Results:** Participants were positive about the product’s potential utility and impact upon confidence in moving around. However, many participants held highly personal perceptions of their vulnerability and need, and expressed concerns about the esthetics and practicality of the accompanying underwear. Participants highlighted potential challenges from poor mobile connectivity, and expressed concerns about product cost.

**Conclusions:** Future research will need to ensure flexible and sensitive approaches to recruitment. Further refinement to the product design may be useful. Individual interviews and questionnaires would help capture participants’ perceptions on personal topics, and measures of changes in confidence. Research sites will need to be compatible with technological functionality. It will be necessary to have a robust protocol in place for withdrawal of the product at the end of any clinical research.

**Implications for Rehabilitation**
- Hip protectors can be effective in preventing hip fractures, but levels of acceptance and adherence may be low and may contribute to low-quality research.
- A new type of hip protector has been designed to overcome some of the acceptance and adherence challenges.
- Older adults suggested that the product was potentially useful, but expressed highly personal concerns about perceived need; aesthetics; practical and technological challenges; and cost, all of which may affect future research design.
- Research designs will need to be flexible enough to consider sensitive approaches to recruitment, multiple methods of data collection, site compatibility with technological functionality, and product withdrawal at end of study.

## Introduction

### The problem

Hip fractures are globally acknowledged as an important public health issue, because of their adverse impacts upon mobility, quality of life and increased health service use [1]. Care of the patient is complex, involving many specialists and agencies; around 30% of patients die within 12 months, and 50% never regain former levels of mobility [2]. Ninety-five percent of hip fractures are caused by falls [3], through a slip, trip, frailty, inadequate protective responses and/or some underlying pathology (e.g., osteoporosis, poor cognition). As the population ages, the incidence of falls is increasing [4]. Those at greatest risk of falls are residents of long-term care facilities [5], people who have had a stroke, hospital inpatients, frail older adults, people with Parkinson’s disease, and people with rheumatoid arthritis [6,7]. In UK, there are approximately 70,000 hip fractures treated every year, at a cost of over £2bn to the health and social care economy [8].

One way to reduce the number of hip fractures may be via the introduction of hip protectors; shields traditionally made of hard plastic or soft foam pads, fitted in specially designed underwear, worn over the greater trochanter to cushion a sideways fall onto the hip. Effectiveness of hip protectors depends on biomechanical performance, and product acceptance (agreement to use hip protectors) and adherence (use in prescribed manner) among users. A recent Cochrane review (19 studies including 17,000 people) has shown that hip protectors can be effective in preventing hip fractures in residents of long-term care facilities when worn at the time of a fall [9]. However, the quality of research is limited by poor study design, small numbers of participants, and lack of information regarding the fall. In particular, key barriers to the implementation of hip protectors are poor levels of acceptance and adherence, which vary widely, and a lack of standardized measures [9]. Reasons for poor acceptance and adherence comprise a range of complex organizational, personal and design factors, including care organization characteristics (e.g., commitment to use, staffing levels), caregiver perceptions, resident clinical profile, product comfort, and effort required to use [9,10]. A better understanding is needed of these factors [9].

### Aims

In this article, we report on a piece of work involving patients and public in the development of research into a new hip protector that aims to overcome some of the challenges relating to poor acceptance, adherence, and lack of information about falls highlighted above. Specifically, we wanted to consult with older adults to inform the design of a feasibility study of the new hip protector, with the ultimate view of progressing to a clinical trial. As part of this consultation, we wanted to examine older adults’ perceptions of the new hip protector, to learn more about factors relating to poor acceptance and adherence that have the potential to undermine recruitment into clinical research.

## Methods

### The hip protector

The technology at the heart of this project was the Fall-Safe Assist hip protector, developed and patented by Hip Impact Protection Ltd, UK, and CE-marked as a class 1 medical device [11]. This technology has two novel elements. First, unlike earlier hip protectors made from soft or hard materials, the Fall-Safe Assist product is made from D30, a soft and malleable material that turns hard upon impact. The hip protectors are worn with specific cotton underwear with pockets to ensure correct placement. Second, the ‘Assist’ aspect of the product refers to an embedded electronic sensor system on a single chip (processor, memory and tri-axial accelerometer, algorithms developed by BioSensics, Boston, MA). This sensor system is linked by Bluetooth Low Energy to an Android mobile/hub, enabling monitoring of:

- direction, speed, and force of fall (via inbuilt accelerometry, height and weight characteristics);
- date, time and location of fall (via existing mobile phone capability) and
- activity prior to the fall (step length and stepping pattern).

The mobile/hub receives these data once an hour from the device. These data are anonymized and held in a secure, encrypted form within a cloud database. The system sends an emergency message to the mobile/hub if at least one of three conditions is met:

1. the wearer does not get up within 20 s after a fall;
2. the wearer’s gait suddenly deteriorates and
3. the level of wearer activity has unexpectedly diminished.

In these conditions, the mobile/hub will send an SMS to a carer or emergency service, including details of the wearer’s location.

### Patient and public involvement in health technology research

The INVOLVE patient and public involvement (PPI) guidelines define PPI as “research… ‘with’ or ‘by’ members of the public rather than ‘to’, ‘about’ or ‘for’ them” [12]. There is increasing recognition of the benefits that PPI can bring to research, including upholding principles inherent to democratic society, and enhancing research relevance and quality [12]. For research into health technologies, the involvement of patients and the public is closely allied with the long-recognized importance of user involvement in technological development [13]. For technologies such as hip protectors that are aimed at older adults, user involvement is particularly important because of the potential mismatch between designers and end-users: technologies are typically designed by younger design teams who may be less familiar with the diversity and needs of older adults, such as living with co-morbid chronic illnesses, adapting to ageing bodies, and different life experiences with technologies [14–16]. Meaningful involvement of older adults in the development of health technologies is important to help avoid a ‘triple loss’, in which older adults do not receive the technologies they need; businesses do not benefit from the so-called ‘silver market’; and public funding continually results in prototypes which are not scaled up [16].

Approaches to PPI include consultation (seeking views), collaboration (ongoing partnership through a project), and user-controlled research (active direction and management by patients and the public) [12]. PPI may inform some or all stages of a research cycle, from initial identification, through designing and undertaking, to evaluating impact of research [12]. In the work reported in this article, we sought consultation with patients and the public to help inform the design of a potential feasibility study of the Fall-Safe Assist hip protectors. We draw upon the short form of the GRIPP2 checklist [17], which offers guidelines for the reporting of patient and public involvement. As per the most recent UK Health Research Authority and INVOLVE statement, application for ethical review was not required [18].

### Participants and consultation events

We conducted five consultation events between April and November 2015, involving older adults and care sector staff in the Midlands and North West of England. The older adults were resident in specialist housing facilities (also known as Sheltered Housing in UK, and Assisted Living Facilities or Supported Living Facilities in USA, Canada and Scandinavia), or were living independently in the community. Some residents in the specialist housing facilities were receiving up to four care visits per day from an on-site care team. The care sector staff were employees of the housing facilities.

In total, we included the views of 147 older adults and 10 care sector staff. The first event was a community falls prevention event with 101 older adults. At this event, we did not capture any demographic information. The remaining older adults and staff took part in one of four smaller events at the housing facilities. At these events we captured information about gender and age distribution. Table 1 shows information about the events and participants.

**Table 1.** Participant details by consultation event.

Event	Older adults	Care sector staff	Gender and age range (older adults)	Gender and age range (staff)
Community falls prevention event	101	0	Not recorded	
Sheltered housing North West 1	11	3	35 female, 11 male, age range 55–96	8 female, 2 male, age range 39–52
Sheltered housing East Midlands 1	9	2		
Sheltered housing East Midlands 2	10	1		
Sheltered housing North West 2	16	4		

The researchers involved were one male (AH) and two females (EB and ES), aged in their 30s and 40s. One of us (ES) is a registered nurse, and collectively we have extensive experience of working with older adults and health and social care staff in research, in the community and in clinical practice, and of health technology development and implementation.

At each event, participants were initially shown examples of hard and soft hip protectors, before seeing the Fall-Safe Assist hip protector, and accompanying underwear. We then asked participants questions relating to their opinions about the Fall-Safe Assist hip protectors as a product, their perceptions upon the potential impact the hip protectors might have upon their health and wellbeing, and their insights into issues of acceptance and adherence that might affect future clinical research (Table 2). These questions were asked in a conversational style, rather than a structured form, to elicit discussion.

**Table 2.** Questions asked during consultation events.

1.	What do you think about the hip protectors, especially with the chip in? What are your feelings about them?
2.	How do you see this as being different from other protective devices and fall alarm systems?
3.	Do you think there might be a place for these hip protectors in places like Specialist Housing facilities for older people, or care homes, or for independently living older adults in the community? What are your thoughts about that? Would it be feasible?
4.	Is there anything that would make these more appealing?
5.	How you think the product could be improved?
6.	How do you think the hip protectors might affect you physically?
7.	If you were to wear the hip protectors, how do you think they might affect your health overall? Your physical, emotional, social, mental health?
8.	Do you think people would be willing to try them out, in a research trial, for three months? Do you think we would be able to recruit people?
9.	What training and support would be needed to maximize the use of these hip protectors in a care setting?

The community falls event was facilitated by ES, who sought responses from participants via an open-ended questionnaire. This event allowed us to get a broad overview of major issues, which we subsequently explored in more detail in the four smaller consultation events that we arranged in partnership with a major provider of supported housing. The first of these smaller events was facilitated by all three researchers, with the initial introduction of the hip protector made by the owner of Hip Impact Protection Ltd. This was so that the older adults could hear how the product had been developed, so that the owner could hear some of their feedback, and so that the three researchers were more clearly informed about the product for future consultation events. The remaining three small consultations were facilitated by AH and EB. At each of these three consultations, EB wore the hip protectors and underwear to demonstrate the fit and appearance underneath clothes. Permission was sought and gained from participants to audio-record the discussions at each consultation, which we later transcribed. A small number of older adults talked to the researchers after the end of the audio recordings. These conversations were written up immediately afterwards. Each event lasted between 1 and 2 h.

## Results

### Perceived need

Many participants talked about how their own, or others’, confidence had been shaken by a fall, and that they could see that wearing the hip protectors might provide reassurance and help them to feel more confident moving around. However, many participants said that they themselves would not wear the hip protectors. Some proffered an innate dislike of any assistive technology, either because it felt unnatural [“it’s not me. It’s not part of me. It’s something else I’m adding to me” (Female, 81)] or because it represented an unwelcome indication of ageing, which “you’ve got to fight” (Female, 70s). A small number of participants thought that the hip protectors would not help them because their falls had been onto their knees, or in another way that had not affected their hips. Others stated that they were not at risk of falls, and had not yet reached the point at which they felt that falls were a concern, e.g., “it would be good for people more vulnerable than me” (Female, 75). When asked about the ‘tipping point’ for agreeing to wear the hip protectors, many participants stated that this would be after their first fall, as esthetic concerns would be overridden by the desire to protect themselves from further physical damage. Some also mentioned that another tipping point might be if people had returned home from hospital. A staff member thought that this point might be an optimal time to encourage use of the hip protectors, as people are often physically weak and need to regain strength and independence.

The majority of participants thought the Fall-Safe Assist system was a good idea. However, once again, when asked if they would use the product themselves, some were less enthusiastic. There were suggestions that the automatic alarm system would work very well for someone else, at high risk of falls. Specific examples given were people living in the community on their own, people who go out walking yet are unsteady on their feet, people in hospital, and people in care homes.

Despite many participants stating that they did not need, or would not use the Fall-Safe Assist hip protectors, at the end of the consultations some participants approached the researchers to ask about trying the product. Staff members also thought that they would have a better chance of encouraging older adults to try the hip protectors on a one-to-one basis, as opposed to in a group setting.

“All the others are saying no, but in a one-to-one situation, I think people are more likely to say ‘I will’” (Female, 52, Staff)

Participants reflected on perceived low levels of understanding about hip fractures amongst their peer group. They suggested that if greater numbers of older adults understood the potential adverse effects of a hip fracture on their physical and mental health and quality of life, more people would likely accept the hip protectors as a preventative measure.

### Aesthetic and practical concerns

Many participants thought that whilst the overall concept of the hip protectors was good, the style of the accompanying underwear was unappealing. At each event, there were some participants who were very negative about the underwear, and thought they would be embarrassed to wear it.

“I’d be embarrassed if I had an accident, or fell outside [and someone saw the underwear]” (Female, 60).

Participants reminded us that increasing age was not necessarily accompanied by disinterest in style of underwear. Each group advocated for choice and variety of different styles of underwear. Some female participants thought that the underwear looked like men’s boxer shorts, and suggested adaptations such as different colors, patterns, lace, or other feminizing factors. Others suggested using different material that could act as ‘control’ underwear, or a ‘girdle’. Some male participants expressed a preference for a ‘fly’ or open front version. The idea of having an open front was discussed at length in two of the groups and was thought to be a good idea for women too, as it would make putting them on and taking them off much easier. A hook and loop fastening was preferred to a button, as manual dexterity was often an issue.

There were also concerns about the compatibility of the hip protectors and underwear with other assistive equipment, such as incontinence pads.

“It might make life a bit more complicated for people that would have to wear [incontinence] pads. I don’t think it would fit in with that” (Female, 50, Staff).

A small number of participants felt that many pairs of underwear would be needed, and that the current offer of four pairs with one set of hip protectors would be insufficient. Some participants had worries about how to wear the protectors correctly, how to wash them, and when to wear them. Staff members thought that cognitive impairment would be a barrier to correct usage, as some people would have to be prompted to wear the protectors.

Some participants suggested that it would be appealing for people if they were able to wear the protectors with their own underwear. To facilitate this, there was some discussion about the development of an adhesive film, which would stick directly to the skin over the hips. However, a small number of participants were concerned about how this might affect older adults with delicate skin.

Linked to the concerns about the underwear were concerns about the size of the hip protector pads. Many participants thought that the pads would be uncomfortable to wear, or would be visible under clothing. At each event, participants made comments such as “they look bulky… if you’re wearing a nice fitted skirt, you’d feel conscious” (Female, 55). However, at events three, four and five, most participants were surprised when it was disclosed that EB was wearing a pair of hip protectors, suggesting that they had not noticed, and that their concerns about visibility were perhaps exaggerated.

### Fall-Safe Assist system: technical concerns

Some participants living in the specialist housing facilities were largely happy with their existing pendant-based alarm system, but could see the potential for wearing the Fall-Safe Assist hip protectors outdoors, where the pendant system did not work. A major concern expressed at all events was the reliance on a mobile phone for the Fall-Safe Assist system to work. Within the specialist housing facilities, the mobile phone and internet reception were reported to be very poor, with text messages sent within and outside the building at times taking over 24 h to be received.

### Cost

At the time of the consultation events, the cost of four pairs of underwear and one set of Fall-Safe hip protector pads without the embedded Assist sensor was £50. Adding in the sensor, to enable use of the Fall-Safe Assist system, increased the cost to £250. The community falls event took place in a fairly affluent area, and cost concerns were not raised by any participants here. However, the four smaller events were held in less affluent areas, and for the majority of participants at these events, the cost of the Fall-Safe Assist system was deemed too expensive, even when taking into account the projected four-year lifespan of the sensor. Some staff members thought that relatives might cover the cost, as it would give them peace of mind, but confirmed that few older adults living in the specialist housing facilities would have the resources to pay for the system. There were some suggestions that the NHS in UK could fund the cost of the underwear and pads, as the alternative cost of care following a hip fracture would be far greater. A small number of participants suggested that older adults could pay in installments. Although any potential participants in future clinical research would not be personally responsible for the cost of the product, some participants suggested that the perception of product cost might render recruitment difficult.

“I’d have a hard job getting him [husband] to wear it for three months. Even free [of charge], I’d have job.” (Female, 81).

There were additional concerns about the associated costs of a compatible mobile phone. None of the participants at the four smaller events reported owning and using a smartphone, and those who owned mobile phones had very basic models.

## Discussion

Hip protectors have the potential to reduce hip fractures, but are beset by poor acceptance and adherence, arising from a range of complex organizational, personal and design factors. In this piece of work, we consulted with older adults and staff of specialist residential care facilities, and in the community, to understand potential issues relating to acceptance and adherence of a new type of hip protector with embedded sensor system, in order to inform the design of future clinical research. Participants offered feedback relating to perceived need, aesthetic and practical concerns, technical concerns, and cost. We discuss the implications of this feedback for future research design.

Feedback from our participants suggests that successful recruitment into clinical trial research may be highly susceptible to potential participants’ own perceptions of their vulnerability and need for the product. If, as many of our participants suggested, the hip protectors would be acceptable to those in more vulnerable situations, further research may best be undertaken with those at high risk of falls and fractures (e.g., in care homes and hospitals), together with frail older adults living in the community. However, participants’ feedback on self-perceived need ranged from the apparently functional (i.e., whether they considered themselves at risk of falls) to the more embodied, biographical (i.e., the perceived impact of the product upon their sense of self). Few of our participants stated (in group settings at least) that they would benefit from using the new hip protector technology. This feedback is congruent with other research findings that older adults did not consider themselves ill enough, or old enough, to need a falls detection technology [19]. The feeling of not being at risk has also been found in the wider research in falls prevention, when considering effective advice [20]. Technologies and devices that medicalize the home, and identify older adults as old and frail, are unpopular [14,16,21,22]. Overall, this feedback suggests that self-perceptions of need are complex and highly personal. This has implications for timings of and approaches to recruitment in any future research. There may be a need for prior education of potential participants about the underlying issue of falls, a need to time an approach at a moment when a potential participant may be more likely to feel in need of the product (e.g., upon discharge from hospital), and to approach participants individually and discreetly. Any clinical research design will therefore require enough flexibility for sensitive approaches to recruitment built into the protocol.

Our participants’ comments also suggested that aesthetic and practical concerns relating to the accompanying underwear would likely present a strong barrier to recruitment. Aesthetics need to be considered so that assistive technology devices are neither noticeable nor identifiable [23,24]. One positive direct outcome of our work is that an adhesive design (developed and tested by 3M^©^ to ensure safety in use with older users who may have fragile skin) has been advanced, so that the Fall-Safe Assist hip protectors could be worn with users’ own underwear. However, further refinement of the product may be necessary before any clinical research could be undertaken with confidence of successful recruitment.

The sensitive, personal nature of perceived need and aesthetic and practical concerns suggest that any future research will need to be mindful of group dynamics, and the possibility of personal discomfort in admitting a need or desire for the product. Future research would likely benefit from the inclusion of individual interviews and questionnaires to capture participants’ views in a manner which would overcome the potential influence of strong voices or embarrassment in group settings. Our participants offered positive comments regarding the potential of the product to increase their confidence in moving around, suggesting that future clinical research should include measures of changes in confidence.

Some of our participants’ comments regarding mobile signal connectivity in their housing facility highlight the need to ensure the suitability of potential research sites to ensure functionality of the technology. They also suggest that further work may need to be done on the product to improve connectivity.

Finally, feedback suggests that some of our participants at the events held in less affluent areas had strong feelings about the perceived cost of the product, even if participants in any future clinical research would not have to pay for the product themselves. The lack of smartphone ownership amongst some participants means that smartphones would need to be costed into any clinical trial. The cost concerns also raise potential ethical issues about the ending of clinical research, in which products that participants may not be able to afford are withdrawn from use. The impact of such withdrawal is under-researched, but may be challenging for researchers and for staff of health and social care facilities in which research is taking place [25]. Any future research design would need a robust protocol in place for withdrawal of the product, and would require clear discussion with potential participants ahead of recruitment. Further PPI work may be useful to refine this aspect of research design.

### Reflections on PPI approach

This work presents an attempt to consult with patients and the public in the early stage of research into a novel hip protector technology, to inform subsequent research design. We acknowledge the presence of strong voices in the consultation events, and the potential for social desirability that may have influenced some participants’ contributions. Upon reflection, a more suitable approach may have been to allocate participants into more suitable sub-groups (e.g., according to gender or history of falls). Our experiences are valuable as they highlight the need for any future research design to be sensitive to the personal nature of the Fall Safe Assist hip protector, and the need for a plurality of methods such as individual interview and questionnaire to capture participant opinions.

Our consultation events took place in specialist housing facilities for older adults, and with community-dwelling adults, therefore perspectives may be restricted to people living within these settings. No care home residents or hospital in-patients were included; these people may have different perspectives regarding the Fall-Safe Assist hip protectors. Nevertheless, it seems likely the main issues our participants raised (perceived need, aesthetic and practical concerns, technological concerns, and issues of cost) would transfer into other contexts.

We recognized that the presence of the company owner had the potential for an overly favourable slant to the presentation of the Fall-Safe Assist hip protectors at the first of the four smaller consultation events. However, we are confident that the remaining three events, at which he was not present, mitigated any such slant.

None of the 46 older adults at the four smaller consultation events owned a smartphone. These participants were therefore not representative of UK average smartphone ownership levels, which are around 50% among 55–64 year olds, and 18% among the over-65s [26]. Nevertheless, the fact that less than one in five UK adults aged over 65 years owns a smartphone means that the concerns raised by our participants are likely to have relevance for future research.

## Conclusions

Participants highlighted issues for future research into the Fall-Safe Assist hip protectors, relating to perceived need, aesthetic and practical concerns, technical concerns, and cost. Future research may best be undertaken with those at high risk of falls and fractures (e.g., in care homes and hospitals), together with frail older adults living in the community. However, recruitment is likely to be challenging, influenced by nuanced self-perceptions of need, and any clinical research design will need to have enough flexibility for sensitive approaches to recruitment built into the protocol. Further refinement of the product design and accompanying underwear may also be necessary. Future research would likely benefit from the inclusion of individual interviews and questionnaires to capture participants’ views on personal topics, and should also include measures of changes in confidence. There will be a need for researchers to consider the compatibility of research sites with functionality of the mobile technology underpinning the ‘Assist’ system. It will be necessary to have a robust protocol in place for withdrawal of the product at the end of any clinical research.

## References

[1] European Union Geriatric Medicine Society A comprehensive fracture prevention strategy in older adults: the European Union Geriatric Medicine Society (EUGMS) statement. Aging Clin Exp Res. 2016;28:797–803. 27299902 10.1007/s40520-016-0588-4

[2] Royal College of Physicians (RCP) National hip fracture database annual report. London: Royal College of Physicians; 2016.

[3] Centers for Disease Control and Prevention (CDC) [Internet] Important facts about falls. Atlanta (GA): Centers for Disease Control and Prevention; [cited 2017 Oct 4]. Available from: https://www.cdc.gov/homeandrecreationalsafety/falls/adultfalls.html

[4] Global Burden of Disease Study Collaborators. Global, regional, and national incidence, prevalence, and years lived with disability for 301 acute and chronic diseases and injuries in 188 countries, 1990–2013: a systematic analysis for the Global Burden of Disease Study. Lancet. 2013;386:743–800. 10.1016/S0140-6736(15)60692-4 PMC4561509 26063472

[5] Ytterstad B The Harstad injury prevention study: the characteristics and distribution of fractures amongst elders-an eight year study. Int J Circumpolar Health. 1999;58:84–95. 10429338

[6] Gillespie LD, Robertson MC, Gillespie WJ, et al. Interventions for preventing falls in older people living in the community (Review). Cochrane Database Syst Rev. 2009;15:CD007146. 10.1002/14651858.CD007146.pub2 19370674

[7] Stanmore EK, Oldham J, Skelton DA, et al. Risk factors for falls in adults with rheumatoid arthritis: a prospective study. Arthritis Care Res. 2013;65:1251–1258. 10.1002/acr.21987 PMC3881513 23436687

[8] National Institute of Health and Care Excellence (NICE) Hip fracture: management. London: National Institute of Health and Care Excellence; 2011. 32073811

[9] Santesso N, Carrasco-Labra A, Brignardello-Petersen R Hip protectors for preventing hip fractures in older people. Cochrane Database Syst Rev. 2014;3:CD001255. 10.1002/14651858.CD001255.pub5 PMC10754476 24687239

[10] Korall AMB, Feldman F, Scott VJ, et al. Facilitators of and barriers to hip protector acceptance and adherence in long-term care facilities: a systematic review. J Am Med Dir Assoc. 2015;16:185–193. 25704127 10.1016/j.jamda.2014.12.004

[11] Hip Impact Protection Ltd. [Internet]. Fall-safe hip protectors. Oxfordshire (UK); [cited 2017 Aug 28]. Available from: http://www.hips-protect.com/

[12] INVOLVE Briefing notes for researchers: involving the public in NHS, public health and social care research. Eastleigh: INVOLVE; 2012.

[13] Damodaran L User involvement in the systems design process – a practical guide for users. Behav Inf Technol. 1996;15:363–377.

[14] Doyle J, Bailey C, Ni, et al. Lessons learned in deploying independent living technologies to older adults’ homes. Univ Access Inf Soc. 2014;13:191–204.

[15] LeRouge C, Ma J, Sneha S, et al. User profiles and personas in the design and development of consumer health technologies. Int J Med Inform. 2013;82:e251–e268. 21481635 10.1016/j.ijmedinf.2011.03.006

[16] Peine A, Faulkner A, Jaeger B, et al. Science, technology and the ‘grand challenge’ of ageing – understand the socio-material constitution of later life. Technol Forecast Soc. 2015;93:1–9.

[17] Staniszewska S, Brett J, Simera I, et al. GRIPP2 reporting checklists: tools to improve reporting of patient and public involvement in research. Res Involv Engagem. 2017;3:13. 29062538 10.1186/s40900-017-0062-2 PMC5611595

[18] Health Research Authority/INVOLVE Public involvement in research and research ethics committee review. Eastleigh: Health Research Authority/INVOLVE; 2016.

[19] Londei ST, Rousseau J, Ducharme F, et al. An intelligent videomonitoring system for fall detection at home: perceptions of elderly people. J Telemed Telecare. 2009;15:383–390. 19948704 10.1258/jtt.2009.090107

[20] Yardley L, Donovan-Hall M, Francis K, et al Older people’s views of advice about falls prevention: a qualitative study. Health Educ Res. 2006;21:508–517. 16467173 10.1093/her/cyh077

[21] Demiris G, Hensel BK Technologies for an aging society: a systematic review of ‘smart home’ applications. Yearb Med Inform. 2008;17:33–40. 18660873

[22] Zwijsen SA, Niemeijer AR, Hertogh CM Ethics of using assistive technology in the care for community-dwelling elderly people: an overview of the literature. Aging Ment Health. 2011;15:419–427. 21500008 10.1080/13607863.2010.543662

[23] Boulton E, Nawaz A, Vereijken B, et al. FARSEEING Deliverable D2.5: updated guidelines for design and implementation of technologies. FARSEEING. 2015.

[24] Hawley-Hague H, Boulton E, Hall A, et al Older adults’ perceptions of technologies aimed at falls prevention, detection or monitoring: a systematic review. Int J Med Inform. 2014;83:416–426. 24798946 10.1016/j.ijmedinf.2014.03.002

[25] Lawton J, White D, Rankin D, et al. Staff experiences of closing out a clinical trial involving withdrawal of treatment: qualitative study. Trials. 2017;18:61. 28173843 10.1186/s13063-017-1813-y PMC5297163

[26] Statista UK [Internet]: smartphone ownership by age from 2012-2017. London: Statista; [cited 2017 August 28]. Available from: https://www.statista.com/statistics/271851/smartphone-owners-in-the-united-kingdom-uk-by-age/

